# Glial Dysfunction and Its Contribution to the Pathogenesis of the Neuronal Ceroid Lipofuscinoses

**DOI:** 10.3389/fneur.2022.886567

**Published:** 2022-04-04

**Authors:** Keigo Takahashi, Hemanth R. Nelvagal, Jenny Lange, Jonathan D. Cooper

**Affiliations:** ^1^Pediatric Storage Disorders Laboratory, Department of Pediatrics, School of Medicine, Washington University in St. Louis, St. Louis, MO, United States; ^2^Department of Pharmacology, School of Pharmacy, University College London, London, United Kingdom; ^3^Zayed Centre for Research into Rare Disease in Children, Great Ormond Street Institute of Child Health, University College London, London, United Kingdom; ^4^Department of Genetics, School of Medicine, Washington University in St. Louis, St. Louis, MO, United States; ^5^Department of Neurology, School of Medicine, Washington University in St. Louis, St. Louis, MO, United States

**Keywords:** Batten disease, neuronal ceroid lipofuscinosis, astrocyte, microglia, oligodendrocyte

## Abstract

While significant efforts have been made in developing pre-clinical treatments for the neuronal ceroid lipofuscinoses (NCLs), many challenges still remain to bring children with NCLs a cure. Devising effective therapeutic strategies for the NCLs will require a better understanding of pathophysiology, but little is known about the mechanisms by which loss of lysosomal proteins causes such devastating neurodegeneration. Research into glial cells including astrocytes, microglia, and oligodendrocytes have revealed many of their critical functions in brain homeostasis and potential contributions to neurodegenerative diseases. Genetically modified mouse models have served as a useful platform to define the disease progression in the central nervous system across NCL subtypes, revealing a wide range of glial responses to disease. The emerging evidence of glial dysfunction questions the traditional “neuron-centric” view of NCLs, and would suggest that directly targeting glia in addition to neurons could lead to better therapeutic outcomes. This review summarizes the most up-to-date understanding of glial pathologies and their contribution to the pathogenesis of NCLs, and highlights some of the associated challenges that require further research.

## Introduction

Lysosomal storage disorders (LSDs) are a group of more than 70 monogenetic diseases characterized by defects in lysosomal metabolism and subsequent accumulation of substrates. Most LSDs present with a broad phenotypic spectrum in multiple organs. This is consistent with the fact that nearly all lysosomal enzymes are ubiquitously expressed and their deficiency will therefore affect many tissue types (1). The neuronal ceroid lipofuscinoses (NCLs or Batten disease) are a group of fatal neurodegenerative LSDs affecting children and young adults. In contrast to other non-neuronopathic LSDs, the NCLs primarily affect the central nervous system (CNS), usually including the retina. The NCLs are remarkably heterogeneous diseases, with studies in both humans and animal models showing that each of 13 subtypes is caused by mutations in different individual genes and have different ages of onset, clinical symptoms, and rate of disease progression (2, 3) (Table 1). As comprehensively reviewed elsewhere (2, 3), a mutation (or mutations) in a different NCL gene causes each form of NCL. Some of these mutations are in soluble lysosomal enzymes (e.g., CLN1, CLN2, CLN10, CLN13), others are in transmembrane proteins within the lysosome (e.g., CLN3, CLN7) or elsewhere in the cell (e.g., CLN6, CLN8), or a range of proteins that vary widely in their nature and location (e.g., CLN4, CLN5, CLN11, CLN12, CLN14).

**Table 1:** Summary of glial changes in mouse models of neuronal ceroids lipofuscinoses.

**Subtype**	**Gene**	**Mouse model**	**Astrocyte activation**	**Microglial activation**	**Oligodendrocytic pathology**	**References**
CLN1	*CLN1/PPT1*	*Ppt1^−/−^*	GFAP+ astrogliosis within spinal cords at 2 months, M1, S1BF, VPM/VPL, LGNd, MGN, CM, and Rt at 3 months, and hippocampus at 7 months.	CD68+ activation within spinal cords at 1 months, F4/80+ activation within M1, S1BF, V1, VPM/VPL, LGNd, and MGN at 5 months and hippocampus at 7 months	Decreased white matter volume in spinal cords at 2–3 months; increased immunoreactivity in Olig2, NG2, and MBP within spinal cords at 1–2 months	(4), (5) (6), (7)
		*Ppt1^Δ*ex*4^*	GFAP+ astrogliosis within cortex at 3 months	F80+ activation within thalamus at 3 months	N/A	(8)
		*Ppt1^*R*151*X*^*	GFAP+ astrogliosis within cortex	CD68+ activation within cortex	N/A	(9)
		*Cln1^*R*151*X*^*	GFAP+ astrogliosis within cortex, thalamus, and hippocampus	CD68+ activation within cortex, thalamus, and hippocampus	N/A	(10)
CLN2	*CLN2/ TPP1*	*Tpp1^−/−^*	GFAP+ astrogliosis within M1 at 2 months and striatum and hippocampus at 3 months	Increase in Iba1 immunoreactivity whithin striatum at 3 months	N/A	(11), (12)
		*Cln2^*R*207*X*^*	GFAP+ astrogliosis within cortex at 3 months	No change in Iba1 immunoreactivity at 3 months	N/A	(13)
CLN3	*CLN3*	*Cln3^Δ*ex*1−6^*	GFAP+ astrogliosis whithin visual cortex, hippocampus, striatum, and cerebellum at 5 months and somatosensory cortex at 7 months	F4/80+ activation within cortex, hippocampus, striatum, and cerebellum at 5 months	N/A	(14), (15)
		*Cln3^Δ*ex*7−8^*	GFAP+ astrogliosis within cortex, striatum, VPM/VPL, and cerebellum at 12 months	F4/80+ astivation within cortex, striatum, VPM/VPL, and cerebellum at 12 months	N/A	(16), (17)
		*Cln3^*Q*352*X*^*	GFAP+ astrogliosis within S1BF and VPM/VPL at 6 months	CD68+ activation within S1BF and VPM/VPL at 6 months	N/A	(18)
CLN4	*CLN4/DNAJC5/CSP*	*Csp^−/−^*	N/A mice die at 2–4 weeks old	N/A mice die at 2–4 weeks old	N/A mice die at 2–4 weeks old	(19)
CLN5	*CLN5*	*Cln5^−/−^*	GFAP+ astrogliosis within S1BF, V1, and VPM/VPL at 1 months and LGNd at 12 months	F4/80+ activation within S1BF, V1, VPM/VPL, and LGNd at 12 months	Reduced MBP+ fibers in S1BF at 1–3 months	(20), (21)
		*Cln5^Δ*ex*3^*	Upregulation of GFAP mRNA in cerebrams at 4.5 months	N/A	Downregulation of MBP and MOG mRNA at 3 months, MAG and PLP mRNA at 4.5 months	(22)
CLN6	*CLN6*	*Cln6^*nclf*^*	GFAP+ astrogliosis within V1, LGNd, and SC at 12 weeks, VPM/VPL and striatum at 21 weeks, and cerebellum at 54 weeks	CD68+ activation within V1, LGNd, and SC at 12 weeks, VPL/VPM, hippocampus, and cerebellum at 54 weeks	N/A	(23), (24)
CLN7	*CLN7/MFSD8*	*Mfsd8^*tm*1*a*/*tm*1*a*^*	GFAP+ astrogliosis within cerebellar white matter at 10 months	CD68+ activation within cerebellum, spinal cord and thalamus at 10 months	N/A	(25)
		*Cln7^−/−^*	GFAP+ astrogliosis within cortex, hippocampus, thalamus, medulla, erebellum, and spinal cord at 5 months	CD68+ activation within cortex, hippocampus, thalamus, medulla, and cerebellum at 7 months	N/A	(26), (27)
CLN8	*CLN8*	*Cln8^*mnd*^*	GFAP+ astrogliosis within VPM/VPL, S1BF, and V1 at 5 months and within LGNd at 8 months	CD68+ activation whithin VPM/VPL, S1BF, V1, and LGNd at 5 months	Decreased white matter volume in corpus callosum and internal capsule at 1–3 months; decreased expression level of MBP and PLP at 1 month; increased G-ration in corpus callosum at 1–4 months	(28), (29)
CLN10	*CTSD*	*Ctsd^−/−^*	Widespread GFAP+ astrogliosis, particularly prominent whithin thalamus and cortex laminae IV-VI at 24 days	CD68+ activation whithin thalamus and substantia nigra at 24 days	N/A	(30)
CLN11	*GRN*	*Grn^−/−^*	GFAP+ astrogliosis within hippocampus, cortex, and thalamus at 24 months	Increased Iba-1 immunoreactivity within hippocampus, cortex, and thalamus at 24 months	N/A	(31)
CLN12	*ATP13A2*	*Atp13a2^−/−^*	GFAP+ astrogliosis within cortex at 1 month, cerebellum, hippocampus, and midbrain at 18 months	N/A	N/A	(32)
CLN13	*CTSF*	*Ctsf^−/−^*	GFAP+ astrogliosis in thalamocortical system at 12 months	F4/80+ microglial activation in thalamocortical system at 12 months	N/A	(33)
CLN14	*KCTD7*	N/A	N/A	N/A	N/A	N/A
CLN15 (proposed)	*TBCK*	N/A	N/A	N/A	N/A	N/A

*M1, primary motor cortex; S1BF, somatosensory cortex barrel field; V1, primary visual cortex; VPM/VPL, medial and lateral ventral posterior nuclei; LGNd, dorsal lateral geniculate nucleus; MGN, medical geniculate; MD, mediodorsal nucleus; CM, central medial thalamic nucleus; Rt, reticular nucleus of thalamus; SC, superior colliculus; GFAP, glial fibrillary associated protein; MBP, myelin basic protein; MOG, myelin oligodendrocyte glycoprotein; MAG, myelin-associate glycoprotein; PLP, proteolipid protein*.

Research into treatments for most LSDs has primarily focused on the replacement of the missing gene product responsible for each disease. Enzyme replacement therapy (ERT) for several soluble enzyme-deficient forms of NCL including CLN1 and CLN2 diseases has been studied (11, 34–38), which led to the recent FDA approval of cerliponase alfa for CLN2 disease (39). However, ERT is only disease-modifying, and several longer-term challenges regarding whether efficacy will be maintained, the delivery systems used and potential immune responses remain (36, 37, 40). Furthermore, ERT is not an option for those subtypes of NCL caused by defects in transmembrane proteins such as CLN3 disease, which is the most common form of NCL (2). Viral vector-mediated gene therapy has been intensively explored as an alternative therapeutic strategy for the NCLs. This approach theoretically has the advantage that a single one-time administration of viral vector should restore deficient lysosomal proteins to transduced cells (41, 42). Preclinical studies of gene therapy in animal models of CLN1, CLN2, CLN3, CLN6, CLN7, and CLN10 diseases have shown promising results (4, 23, 34, 43–47). However, clinical studies in children with CLN2 disease treated with gene therapy showed considerably less efficacy (48, 49), highlighting the difficulty of translating advances from mice directly into human patients (50). Indeed, none of the therapies that are currently available or being tested clinically are curative. Therefore, devising optimal therapeutic strategies for the NCLs will certainly require a better understanding of pathophysiology in each form of NCL.

Neuropathology in the NCLs was initially characterized in human autopsy studies, revealing marked neuron loss accompanied by intra-lysosomal accumulation of autofluorescent storage material (AFSM), whose major protein component is subunit C of mitochondrial ATP synthase (SCMAS), in addition to astrogliosis, and microglial activation (51, 52). With the limited availability of genetically validated human autopsy samples, many longitudinal studies in animal models have been performed, in order to understand the staging of neuropathological processes from the earliest events to the end-stage of disease. Interestingly, AFSM accumulation, astrogliosis, microglial activation and neuron loss in animal models of NCL are remarkably selective in their early stages, becoming more widespread with disease progression (53). This suggests that despite the ubiquitous expression of these proteins, such selective vulnerability may be due to them playing physiological roles of greater importance in some cell populations than others.

A significant finding made in multiple mouse models across subtypes of NCL is the profound loss of thalamic neurons, which typically precedes neuron loss in the corresponding region of the cortex to which these thalamic neurons relay (5, 14, 16, 17, 20, 28, 30, 54). Strikingly, these studies in mouse models also revealed that localized astrocytic and microglial activation, which both occur early in disease progression, accurately predict where subsequent selective neuron loss occurs in mouse models of a majority of NCL subtypes. Such findings cast doubt on traditional perspectives of the NCLs as predominantly “neuronal” diseases, and lead to the hypothesis that abnormalities in glial cells may contribute to the neurodegeneration associated with the NCLs.

In the “neuron-centric” past of neuroscience, glial cells were often relegated to being considered as undefined passive structural elements, and in the diseased state glial activation was often considered a secondary response to neuron dysfunction or damage. Over recent decades, this traditional neuron-centric conception of the CNS has been challenged by a large body of research aiming to provide a better understanding of glial function, revealing that glial cells including astrocytes, microglia, and oligodendrocytes have more active roles in both neuronal homeostasis and neurodegeneration (55–57). Notably, recent technological advancements have enabled us to study the heterogeneity of each glial cell type, and have revealed their bimodal or multimodal roles in neurodegenerative diseases (58, 59). This review aims to summarize the recent progress in our understanding of glial pathologies and their contribution to NCL pathogenesis and examines where NCL research currently stands in the field of glial biology. This review focusses primarily upon CLN1, CLN2 and CLN3 diseases as the three most common forms of NCL, in which a consideration of glial dysfunction or the contribution to pathogenesis has been undertaken or is underway. However, where available, the extent of astrogliosis and microglial activation or oligodendrocyte pathology is listed in mouse models of other forms of NCL in (Table 1). As discussed below, these immunohistochemically detectable changes may be due to dysfunction of glial cell types (which is largely unexplored in most NCLs), or reflect their response to ongoing neuronal dysfunction or loss.

## Glial Dysfunction in the NCLs

### Astrocytes

Neuroimmune responses mediated by both astrocytes and microglia have crucial roles in all CNS insults including brain injury, infection, and neurodegenerative diseases (60, 61). In response to these insults, astrocytes and microglia become “activated” or “reactive” by altering their morphology, protein expression, and secretion profile. The fact that astrocytes and microglia typically both become activated in concert has made it difficult to distinguish the relative contributions of astrocytes to neurodegeneration, and whether these are distinct from those of microglia. Nonetheless, understanding their distinct patterns of activation in disease states is very important.

Upregulation of intermediate filaments, most notably glial fibrillary acidic protein (GFAP), is a classic marker for astrogliosis in mammalian models, and the expression level of GFAP or immunohistochemical detection of this marker has proved a useful tool to assess the extent of astrogliosis (62). As summarized in Table 1, GFAP-positive astrogliosis has been documented in all characterized mouse models of NCL. Although astrogliosis is observed in multiple CNS regions toward the end stage of disease, typical astrogliosis in the NCLs is characterized by its regional specificity and timing; astrogliosis especially occurs early and is pronounced in thalamocortical pathways where considerable subsequent neuron loss occurs (reviewed in 26, see individual references in Table 1). This is in contrast to many other neuropathic LSDs such as mucopolysaccharidosis (MPS), in which astrogliosis tends to be more generalized across the CNS throughout disease progression (63, 64). Interestingly, the extent of GFAP reactivity and morphological alteration in astrocytes varies across the NCLs. For example, hypertrophy of astrocyte cell bodies and processes and GFAP upregulation in *Cln*3^−/−^ mice appears to be more subtle or perhaps attenuated compared to astrocytes observed in *Ppt*1^−/−^ mice (14), implying that CLN1 and CLN3 diseases differ in the extent to which astrocytes are intrinsically dysfunctional and/or respond to extracellular stimuli. These differences in astrogliosis in CLN1 and CLN3 diseases are also recapitulated by *in vitro* experiments using primary astrocytes derived from the relevant mouse models; *Ppt*1^−/−^ astrocytes exhibit a more activated morphology and higher expression levels of GFAP, and enhanced secretion of cytokine and chemokine compared with the wild-type astrocytes (65). In contrast, *Cln*3^−/−^ astrocytes showed attenuated changes in morphology and GFAP expression in response to pharmacological stimulation with reduced secretion of a range of neuroprotective factors, mitogens, cytokines, and chemokines (66). It will therefore be important to further investigate the nature of astrocytic dysfunction using similar tissue culture methods for other forms of NCL such as CLN2 disease.

Recently, it has been demonstrated that GFAP depalmitoylation is regulated by PPT1, and blocking palmitoylation by the unique palmitoylated residue in GFAP attenuates both astrogliosis and the concurrent neurodegenerative pathology in CLN1 mice (67). This is the first evidence suggesting that loss of NCL proteins in astrocytes directly impacts an intrinsic astrocyte response rather than “reactive astrogliosis” occurring solely in response to ongoing neuronal damage. However, these findings appear somewhat contradictory to previous evidence showing that prevention of GFAP upregulation by knocking out both GFAP and Vimentin in *Ppt*1^−/−^ mice (*Gfap*^−/−^; *Vimentin*^−/−^; *Ppt*1^−/−^) exacerbates disease pathology, which had been interpreted as evidence for a protective role of GFAP upregulation in CLN1 disease (68). Not only do such findings imply multi-dimensional roles of astrogliosis, which will be discussed shortly, but also potentially different pathological impacts depending on NCL subtype, affected brain regions and staging of disease progression.

Recent efforts have focused on gene expression profiling of activated astrocytes both *in vitro* and *in vivo* to decipher their functional properties in the context of neurodegeneration. The paradigm of neurotoxic “A1” astrocytes and neuroprotective “A2” astrocytes is now a generally recognized concept (62, 69). Astrocytes resembling “A1” or neurotoxic status have been reported in more common neurodegenerative diseases such as Alzheimer's disease (AD) (70), amyotrophic lateral sclerosis (ALS) (71), and Parkinson's disease (72). Similarly, the pronounced typical A1-specific molecular signature has been recently reported in the forebrains of *Ppt*1^−/−^ mice (73), suggesting a neurotoxic function of astrocytes in CLN1 disease. However, caution is needed in using the current A1/A2 classifications to interpret pathological roles of astrocytes, because such a binary A1/A2 paradigm may be an oversimplification of potentially more wide-ranging and heterogeneous states of astrogliosis (74). Indeed, the recent RNA sequencing data of *Tpp*1^−/−^ mice have shown changes in the expression of a restricted subset of A1- or A2-specific genes, which does not match the typical A1/A2 classification (75). A lack of clear A1/A2 signature has also been reported in other diseases including Huntington disease (76), highlighting that astrocyte heterogeneity may convolute A1/A2 boundaries. Nevertheless, there is a potential that these widely accepted A1/A2 markers can still be useful for both investigating the pathological contribution of astrogliosis, comparing astrocyte phenotypes in the NCLs to other neurodegenerative conditions and assessing the efficacy of therapeutic approaches for NCLs.

Astrocytes also exert pathological influences on neuronal health through multiple non-inflammatory functions such as neurotransmitter recycling, ion buffering, and the release of growth factors (77, 78). In addition, the role of phagocytosis by astrocytes in synaptic connectivity is now in the spotlight but has been relatively understudied in neurodegenerative diseases (79). Considering their close relationship with lysosomal calcium signaling and lysosomal exocytosis, it is plausibly speculated that the loss of NCL proteins could affect many of these non-inflammatory functions of astrocytes. Impaired calcium signaling in primary astrocytes derived from *Ppt*1^−/−^ and *Cln*3^−/−^ mice has been documented (65, 66). Therefore, it will be important to decipher the molecular bases of possibly more diverse forms of astrocytic dysfunction in the NCLs rather than solely focusing on astrogliosis to better understand the pathological role of astrocytes in NCL pathogenesis.

### Microglia

Microglia, the CNS tissue resident macrophage population, also become “activated” or “reactive” by changing their gene expression, morphology, motility, migration, metabolism, secretome, phagocytosis, proliferation, and death in response to CNS pathology (61). Microglial-astrocyte crosstalk via the release of diverse signaling molecules is particularly thought to mediate neurodegeneration (80), with recent studies suggesting that neurotoxic A1 astrocytes are triggered by fragmented mitochondria released from microglia to propagate and trigger neuronal death (81, 82).

Classically, immunoreactivity of several molecular markers including CD68, MHC antigen class II, F4/80, and Iba1 have been widely used to define the activated state of microglia (83, 84). Longitudinal studies using several of these markers have confirmed that where examined microglial activation is invariably present in the CNS of NCL mouse models, and anatomical distribution and onset of microglial activation largely overlap those of astrogliosis (Table 1). Although comprehensive profiling of multiple microglial markers is still underway, data so far suggest that the nature of microglial activation appears to be different in each NCL. This subtype-dependent nature of microglial activation is buttressed by *in vitro* primary culture experiments in CLN1 and CLN3 disease; *Ppt*1^−/−^ microglia are morphologically more activated with increased secretion of IL-1β (65), whereas *Cln*3^−/−^ microglia exhibit attenuated morphological responses to pharmacological stimulation with reduced secretion of several chemokines (66). Notably, when *Ppt*1^−/−^ astrocytes and microglia were co-cultured, they appeared to cross-prime one another to exacerbate neuron loss (65), implicating the involvement of astrocyte-microglia crosstalk in CLN1 disease pathophysiology.

Recent research has been delineating the complex and heterogeneous state of activated microglia, a topic that is still under debate. The classification of pro-inflammatory “M1” microglia vs. anti-inflammatory “M2” microglia using the expression of particular cell surface markers and cytokines had been long recognized (57, 84), despite the validity of such a classification still being under scrutiny. M1 polarization of microglia with upregulation of *CD16/32* and *CD86* has been reported in *Ppt*1^−/−^ and *Cln*3^−/−^ mice, and knocking out of the inflammation-related cell adhesion molecule sialoadhesin in those mice attenuated numbers of M1-polarized microglia, levels of pro-inflammatory cytokines, and altered disease phenotype (85). However, given criticism that the M1/M2 dichotomy provides an oversimplified perspective (86, 87), a new pathological classification that incorporates the concept of disease-associated microglia (DAM) has recently been put forth (58, 88). DAM are molecularly characterized by the expression of typical microglial genes such as *Iba1, Cst3*, and *Hexb*, coincident with downregulation of homeostatic microglial genes including *P2ry12, P2ry13, Cx3cr1, CD33*, and *Tmem119* (89). DAM further display upregulation of genes involved in lysosomal, phagocytic, and lipid metabolism pathways such as *Apoe, Ctsd, Lpl, Tyrobp*, and *Trem2*, which perhaps makes the DAM classification particularly pertinent to LSDs. RNA sequencing data has revealed the existence of both TREM2-independent and TREM2-dependent DAM in *Tpp*1^−/−^ mice, suggesting the pro-inflammatory and neurotoxic role of activated microglia in CLN2 disease (75, 90). However, the pathological role of DAM still remains debatable; several recent studies have shown neuroprotective effects of TREM2-dependent DAM in mouse models of AD and GRN haploinsufficiency-causing frontotemporal lobar degeneration (GRN-FTLD) (91, 92), suggesting the pathological contribution of DAM may well be disease-dependent. Interestingly, complete deficiency of *Grn*^−/−^ is known to cause CLN11 disease (31), suggesting a similar phenotype may exist in some forms of NCL. Therefore, caution should be exercised in overinterpreting data for the expression of, or staining for, DAM markers and it will be wise not to solely rely on such findings when interpreting pathological roles of activated microglia in NCL pathogenesis in future studies.

The secretion of cytokines and chemokines is of paramount importance for both astrocytes and microglia to exert pro- and anti-inflammatory effects on the process of neurodegeneration (93). The progressive elevation of multiple cytokines and chemokines has been confirmed by whole transcriptomics and/or proteomics in the forebrains and cerebella of *Tpp*1^−/−^ mice (75, 90) and forebrains and spinal cords of *Ppt*1^−/−^ mice (68, 94, 95). Such evidence for the region- and subtype-specific nature of neuroinflammatory changes in CLN1 and CLN2 diseases correlates with the previously shown region- and subtype-specific immunoreactivity of astrogliosis and microglial activation markers. Pharmacological modulation of neuroinflammation is an emerging therapeutic strategy for neurodegenerative diseases (96). Until now, only a few anti-inflammatory drugs have been preclinically tested for NCLs: fingolimod, teriflunomide, and MW151 in *Ppt*1^−/−^ mice (97, 98) and ibuprofen and mycophenolate motefil in *Cln*3^−/−^ deficient mice (99, 100) and provide only partial phenotypic rescue. While modulation of neuroinflammation may provide additional therapeutic benefit, especially when used in combination with other therapies such as ERT or gene therapy, these preclinical results suggest that alteration of central pro-inflammatory cascades in NCL mice might be a non-specific downstream consequence.

Other non-immune-related properties of microglia also have a significant impact on neuronal health. Microglial-mediated phagocytosis is critical in maintaining CNS homeostasis by pruning synapses or phagocytizing dysfunctional, dying or the debris of deceased neurons and other cell types (57, 101). It has been shown that impaired microglial phagocytic function promotes the development of several neurological diseases such as Rett syndrome (102) and tuberous sclerosis complex (103). Since phagocytosis requires focal exocytosis of lysosomes (104), it is plausible to speculate that lysosomal dysfunction due to NCL protein deficiency could also impair phagocytosis in these cells. While there have been several pieces of evidence from RNA sequencing or proteomics analysis suggesting altered phagocytosis in the brains of *Ppt*1^−/−^ and *Tpp*1^−/−^ mice (75, 94), microglial-specific alteration of phagocytosis is yet to be elucidated. A better understanding of the nature of such dysfunctional phagocytosis by microglia and its contribution to NCL pathogenesis may therefore inform us of new therapeutic targets.

### Oligodendrocytes and Schwann Cells

Demyelination is another pathological change widely seen in multiple neurodegenerative diseases. Consistent with recent evidence suggesting the regulatory roles of lysosomal exocytosis in myelination, abnormal myelination is commonly seen in many LSDs including Niemann-Pick disease, Gaucher disease, metachromatic leukodystrophy, multiple sulfatase deficiency, and Krabbe disease (105–107). In contrast, pathological evidence of either dysmyelination or demyelination in the NCLs has been investigated only in mouse models of CLN1, CLN5, and CLN8 diseases with limited depth of characterization (Table 1). A key question is whether overt demyelination occurs at all in these disorders, or whether any changes in myelin composition occur secondary to loss of axons, as a result of neuron loss. Certainly, changes in white matter volume are evident in both animal models and human autopsy specimens (6, 21, 29), but its basis is poorly understood. Of course, any consideration of myelin must necessarily include Schwann cells in the peripheral nervous system (PNS), which serve a similar, but not identical role to oligodendrocytes in the CNS. However, the pathological impact of the NCLs upon the PNS is largely underappreciated, but is currently of renewed interest.

## Contribution of Glia to NCL Pathogenesis

A key question that remains to be answered is whether or not the loss of NCL proteins from glial cells confers any direct cell-autonomous effects on these glial cells themselves and/or non-cell-autonomous effects on other cell types including neurons in either a harmful or protective manner. In *in vitro* studies using primary astrocytes, neuron-glial co-culture experiments showed that both *Ppt*1^−/−^ and *Cln*3^−/−^ glia are detrimental to the survival of both wild-type and mutant neurons (65, 66). Such data raise the possibility that mutant astrocytes and microglia may actively trigger the neurodegenerative changes seen in CLN1 and CLN3 diseases. Such *in vitro* models are a crucial component in unraveling cell-type-specific contributions to disease pathogenesis and lend themselves to high throughput screening to detect novel phenotypes and assess potential therapeutic interventions (108–110). Using this approach has highlighted disease-modifying pathways in a number of neurodegenerative diseases that may provide valuable therapeutic targets. Furthermore, the advent of induced pluripotent stem cell (iPSC) models allows the close physiological representation of disease-affected cells on a species-specific genetic background. iPSC models have only been used to a limited extent in the NCLs to date and have so far not been used to generate glial cells despite the availability of well-established differentiation protocols (111–113). For the NCLs, it will be vital to further investigate glial phenotypes *in vitro* and to validate those findings by generating cell-type-specific mutant mice to explore these issues *in vivo*.

Microglial depletion using CSF-1R inhibitors has enabled us to study the direct effect of microglia on the CNS disease process in mammalian models (114). With this technique, it has been shown that microglial depletion in *Ppt*1^−/−^ mice attenuated optic nerve pathologies and several behavioral abnormalities (115). Although such findings might be confounded by the fact that completely abolishing microglia is likely to negatively impact CNS homeostasis, such studies still provide a degree of mechanistic insight into microglial contributions to CLN1 disease progression. Since the effectiveness and safety of some CSF-1R inhibitors have been proven in humans (114) and as new and more specific CSF-1R inhibitors become available, microglial depletion may be a clinically relevant approach.

The *Cre-LoxP* system in mice has proved a powerful tool to investigate the effect of cell-type-specific genetic mutation on neurodegeneration and applied to a wide range of diseases including LSDs *in vivo*. For example, it has been shown that astrocytic-specific deletion of Sulfatase Modifying Factor 1 (SUMF1) (*Sumf1*^*flox*/*flox*^; *GFAP-Cre*) was sufficient to induce neuron loss in a mouse model of multiple sulfatase deficiency (MSD) (116). Also, microglial-specific deletion of NPC1 (*Npc1*^*flox*/−^; *Cx3cr1-Cre*) has been shown to enhance microglial phagocytotic uptake and impaired lipid trafficking, resulting in impaired myelin turnover in a mouse model of Niemann-Pick type C (NPC) disease (117), caused by a deficiency in the NPC1 protein. In contrast, it has also been shown that astrocytic-specific deletion of NPC1 (*Npc1*^*flox*/−^; *GFAP-CreER*) does not cause neurodegeneration, but neuron-specific knockout (*Npc1*^*flox*/−^; *Syn1-Cre*) does in the NPC mouse model (118). Such data suggest that the nature of the glial contribution to pathogenesis is likely to differ between LSDs. However, no study has yet investigated the effect of astrocyte-, microglial-, or oligodendrocyte-specific deletion of NCL genes *in vivo* has been reported, indicating that NCL research regarding glial pathology is admittedly lagging behind other LSDs. Perhaps this is in part because of the sheer body of work this would entail given the number of NCL subtypes, as well as the fact that several of the genes that are deficient in the NCLs are lysosomal enzymes that are normally secreted and can be taken up by neighboring cells via a variety of receptor subtypes (42). This process of “cross-correction” naturally confounds and complicates any attempts to generate cell-type-specific PPT1 or TPP-1 deficient mice. However, recent work in creating chimeric “tethered” versions of enzymes might indeed enable the creation of conditional cell-type-specific models (119).

## Conclusions and Future Directions

Our relatively poor understanding of the pathomechanisms that operate in the NCLs has certainly hampered the generation of more effective therapeutic strategies. Until recently, glial cells across various neurodegenerative diseases have often been considered as poorly defined passive structural elements. The underappreciated consideration of glial involvement in the NCLs is no exception, which is perhaps reflected by the re-naming of these disorders in the 1960s as “neuronal ceroid lipofuscinoses” (120) to distinguish them from other childhood encephalopathies. The rapidly expanding body of research into normal glial biology and their responses to disease has facilitated a reassessment that glia are not just passive bystanders of pathology in the CNS, but instead are active determinants of neurodegeneration. As summarized in this review, there is substantial evidence suggesting such glial involvement in NCL pathophysiology, and changes in glial activation are frequently used to evaluate therapeutic efficacy in preclinical studies (4, 11, 15, 23, 26, 34, 43–47, 97). Of necessity, this review focusses primarily upon the three most common forms of NCL, CLN1 disease, CLN2 disease and CLN3 disease, in which the issue of glial contribution to pathogenesis has been considered. Nevertheless, as detailed in Table 1, glial activation is present in all forms of NCL and is consistently present before neuron loss occurs. As such, we might anticipate that glia may also be involved in the pathogenesis of these other forms of NCL. However, given the pronounced difference between even CLN1, CLN2 and CLN3 disease that are discussed in this review, it could be expected that the extent and nature of glial involvement may also vary markedly between types of NCL. Nevertheless, although the glial contribution to disease progression has been intensively studied in other neurodegenerative diseases, relatively little is known about whether glia contribute mechanistically to the profoundly neurodegenerative phenotype of most forms of NCL.

There are several remaining issues that still need addressing in order to clarify the contribution of glial pathology in the NCLs. First, all of the many of subcellular alterations known to be associated with NCLs and other LSDs such as impaired autophagy, lysosomal trafficking, and alterations in the mTOR and TFEB signaling pathways have primarily been studied in neurons or fibroblasts, but not specifically in glial cells of any variety (27, 104, 121–126). Indeed, there is considerable potential that studying these pathways in NCL glia will yield valuable mechanistic information about cell-type-specific impacts of disease-causing mutations. Second, while NCL research has predominantly relied on mouse models, recent evidence has suggested species-dependent differences in the functional properties of astrocytes, questioning the translational relevance of information mouse astrocytes (127). As this issue almost certainly applies to microglia and oligodendrocytes as well, the implementation of glia differentiated from human NCL-patient-derived iPSCs is likely to be of considerable benefit (113). Third, as already discussed, studying the cell-autonomous effects of soluble enzyme deficiency *in vivo* is hampered by “cross-correction,” a phenomenon via which mannose 6-phosphate receptor-mediated endocytosis facilitates extracellularly delivered lysosomal enzymes to be taken up by recipient cells. As a previous example of the way to overcome this challenge, the chimeric GALC enzyme tethered to the lysosomal membrane has been engineered in the Krabbe disease mouse model so the cell-autonomous effect of oligodendrocyte-specific GALC deficiency could be studied (119). It will be important to extend such methodology to PPT1 and TPP1 in order to address the cellular autonomy of CLN1 and CLN2 diseases, respectively.

Modern “omics technologies have greatly contributed to a better understanding of the complex physiological nature of glial pathologies in the NCLs and other LSDs (128, 129). RNA sequencing has been widely used in the field of NCLs now that its cost is substantially reduced, but there are a number of caveats concerning the validity of RNA sequencing results. For example, RNA sequencing of a bulk tissue cannot distinguish molecular events in different cell types. As such distinct molecular changes that occur in specific glial cell populations such as microglia and oligodendrocytes, which comprise a relatively small proportion of the total cells present in these samples, might be masked. The application of the single-cell or single-nucleus RNA sequencing technology can theoretically overcome this issue (101), and is likely to reveal new insights into the broad range of effects upon glia in the NCLs. Another issue, which is perhaps unique in LSD research, is that lysosomal proteins play a crucial role in post-translational modification and intracellular trafficking (104, 130), which transcriptomics analyses cannot address. Proteomics analysis instead is more suitable in this case, but again, proteomic data obtained from bulk tissue cannot distinguish between different cell types. Most recently, single-cell proteomics technologies have been invented (131), and it may be predicted that this approach will be widely used to study glial biology in near future.

Notably, glia also exist outside the CNS in different forms depending on the anatomical region. Schwann cells are the myelinating cells in the peripheral nervous system (PNS) and are involved in maintaining ionic balance and providing support to axons (132). There are also non-myelinating Schwann cells called terminal Schwann cells, residing at the neuromuscular junction (133). Satellite glial cells are found in peripheral ganglia and potentially have similar functions to astrocytes in the CNS (134). There is also a unique population of astrocyte-like cells called enteric glial cells, involved in the regulation of the intestinal epithelial barrier and in regulating the function of neurons within the enteric nervous system (ENS) (135). Given the accumulated evidence for glial abnormalities across multiple forms of NCL, it will be important to investigate the impact of disease upon these “non-CNS glial cells” that are key components of the PNS and ENS. These may represent important cellular targets to obtain better therapeutic outcomes in patients with NCLs.

To conclude, much like the different types of musicians in a band that need to coordinate together with its singer to produce harmonious music, different glial cells provide coordinated support for neuronal health. As in a band it only takes one member to perform sub-optimally for the music to be compromised, and it is very likely that the dysfunction of any one type (or types) of glia similarly contribute to neurodegeneration. With recent technical advances, we are now entering an exciting time for expanding our knowledge of glial dysfunction and its contribution to the pathogenesis of the NCLs. This knowledge will almost certainly help us design more effective and appropriately targeted therapeutic strategies for these disorders.

## Author Contributions

KT: conceptualization, investigation, and writing the original draft. HN and JL: writing, reviewing, and editing. JC: supervision, conceptualization, reviewing, and editing. All authors contributed to the article and approved the submitted version.

## Funding

The many studies from the Pediatric Storage Disorders Laboratory (PSDL) cited in this review were funded from a variety of sources: US National Institutes of Health (NS 043205, NS41930, NS44310, NS116574, and NS117635), the Wellcome Trust, UK Medical Research Council, European Union 6th Framework Programme, European Union 7th Framework Programme and Horizon 2020 research and innovation programme under Grant Agreement No. 666918 (BATCure), Sparks Foundation, Batten Disease Support and Research Association (BDSRA), Batten Disease Family Association (BDFA), Beyond Batten Disease Foundation, NCL Stiftung, The Saoirse Foundation and Health Research Board of Ireland, Noah's Hope/Hope for Bridget, The Fore Batten Foundation, Hailey's Heroes, The Children's Brain Diseases Foundation, The Bletsoe Family, and The Natalie Fund. This work was also made possible by institutional support from the Department of Pediatrics, Washington University in St Louis to JC, and a McDonnell International Scholars Academy award to KT.

## Conflict of Interest

JC has received research support from BioMarin Pharmaceutical Inc., Abeona Therapeutics Inc., Regenexbio Inc., and Neurogene. The remaining authors declare that the research was conducted in the absence of any commercial orfinancial relationships that could be construed as a potential conflict of interest.

## Publisher's Note

All claims expressed in this article are solely those of the authors and do not necessarily represent those of their affiliated organizations, or those of the publisher, the editors and the reviewers. Any product that may be evaluated in this article, or claim that may be made by its manufacturer, is not guaranteed or endorsed by the publisher.
